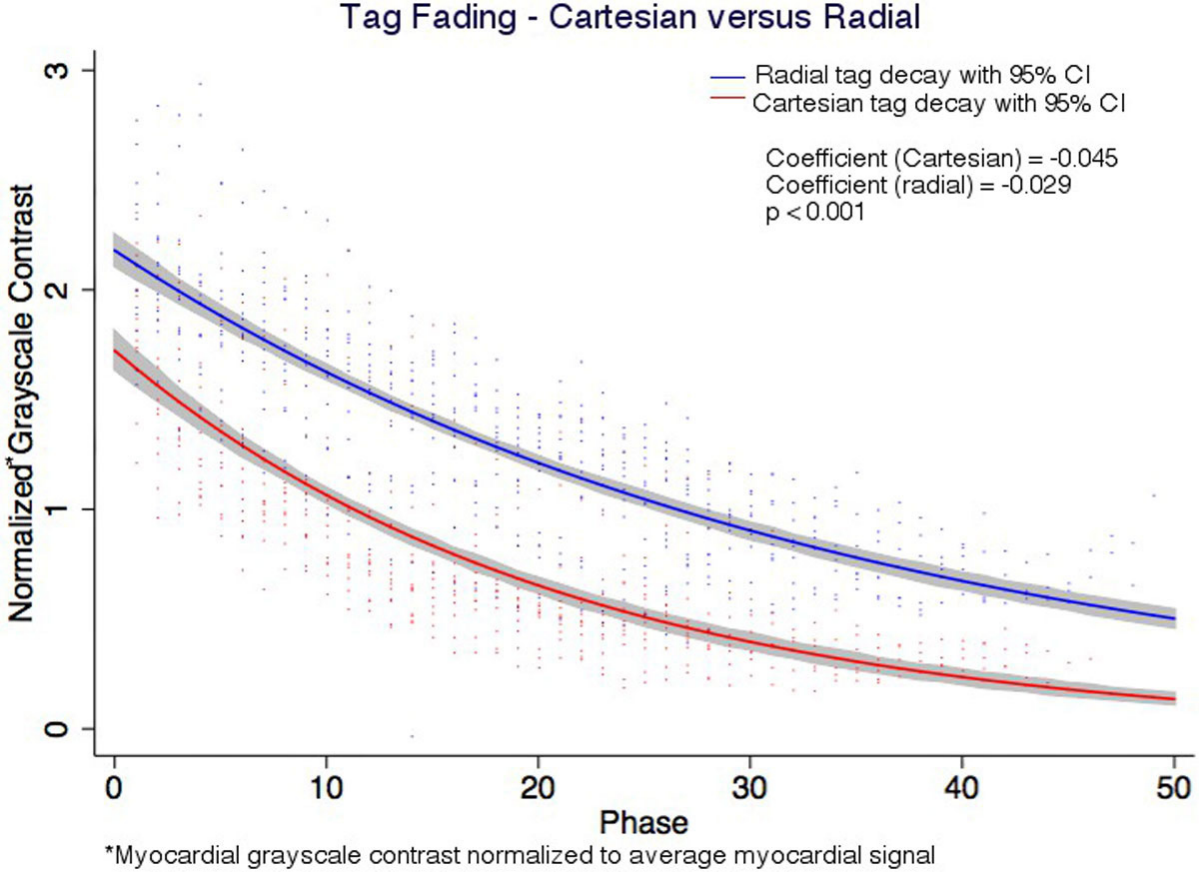# Myocardial strain imaging with radial acquisitions (SIRA) reduces tag fading compared to Cartesian sampling

**DOI:** 10.1186/1532-429X-16-S1-P35

**Published:** 2014-01-16

**Authors:** Edward Hulten, Ravi V Shah, Siddique Abbasi, Tomas Neilan, Jiazuo Feng, John Groarke, Alfonso H Waller, Ron Blankstein, Raymond Y Kwong, Michael Jerosch-Herold

**Affiliations:** 1 Noninvasive Cardiovascular Imaging, Brigham and Women's Hospital and Harvard Medical School, Boston, Massachusetts, USA; 2 Division of Cardiology, Massachusetts General Hospital and Harvard Medical School, Boston, Massachusetts, USA; 3 Cardiology, Walter Reed National Military Medical Center, Bethesda, Maryland, USA

## Background

Myocardial tagging is considered a gold-standard technique to derive myocardial strain using cardiac magnetic resonance (CMR) in large epidemiologic studies. Nevertheless technical limitations related to tag line fading (via T1 relaxation) limit generalizability and reproducibility of this technique. Our purpose was to test myocardial tagging with radial k-space acquisition against routinely utilized Cartesian k-space sampling techniques, in order to potentially minimize tag-line fading over the cardiac cycle.

## Methods

We compared a new tagging sequence with radial acquisitions for cardiac MRI to a routinely-utilized clinical tagging sequence with Cartesian sampling. Both sequences were added pre-contrast to routine clinical scans of 14 patients. The per phase change in grayscale contrast due to T1 relaxation ("tag fading") of tagged myocardium was modeled using a non-linear fit with maximum likelihood estimation to an exponential decay curve and compared between groups. Peak Eulerian circumferential strain (Ecc) was compared using HARP software.

## Results

14 patients (mean age 54 ± 16 years and 36% were men) and mean left ventricular ejection fraction 53 ± 16% (LVEF) were included. Tag fading differed significantly between radial and Cartesian sampling; the exponential decay coefficient was -0.045 ± 0.011 for the Cartesian and significantly higher than the-decay coefficient value of 0.029 ± 0.007 for radial read-outs (p < 0.001). Due to less tag fading myocardial strain could be evaluated for more segments acquired with radial, compared to Cartesian read-outs (p < 0.001). The intra-observer reliability for Ecc was higher for radial (r = 0.99, p < 0.001) than Cartesian (r = 0.86, p=<0.001) images (p = 0.002 for comparison). The bias was 0.9% and limits of agreement -4.1 to 6.0% with concordance = 0.86 (p < 0.001) for Ecc-radial versus Ecc-Cartesian. Both tagging methods correlated with LVEF, r = -0.86 (p < 0.001) for Ecc-radial versus r = -0.83 (p < 0.001) for Ecc-Cartesian.

## Conclusions

Radial k-space acquisition with very low flip-angle excitations leads to better reliability for myocardial strain relative to standard, widely employed Cartesian readouts, while maintaining good signal-to-noise. Radial k-space acquisition is a promising new approach for evaluating myocardial strains using MRI.

## Funding

None.

**Figure 1:**